# Determination of the most important risk factors of gestational diabetes in Iran by group analytical hierarchy process 

**Published:** 2017-02

**Authors:** Masoumeh Abbasi, Zahra Mazloum Khorasani, Kobra Etminani, Rasool Rahmanvand

**Affiliations:** 1 *Research Committee, Department of Medical Informatics, School of Medicine, Mashhad University of Medical Sciences, Mashhad, Iran.*; 2 *Endocrine Research Center, Mashhad University of Medical Sciences, Mashhad, Iran.*; 3 *Department of Medical Informatics, School of Medicine, Mashhad University of Medical Sciences, Mashhad, Iran.*; 4 *Islamic Azad University, South Tehran Branch, Tehran, Iran.*

**Keywords:** Gestational diabetes, Decision making, Risk factors

## Abstract

**Background:**

The outbreak of gestational diabetes has a significant increase during recent years. This disease has complications for mother and her baby. Screening is an opportunity for preventing of gestational diabetes complications.

**Objective:**

The aim of this research was to determine the most important risk factors for Gestational Diabetes Mellitus (GDM) in Iran according to the expert's views by Group Analytical Hierarchy Process.

**Materials and Methods:**

In this cross-sectional study, papers related to the prevalence and risk factors of GDM in Iran from 1992-2015 were reviewed. By studying texts and Up to Date databases, 10 risk factors for gestational diabetes were collected. Among these 10 items, the risk factors that have become significant based on studying literature in Iran were selected for analysis. Group Analytical Hierarchy Process (GAHP) questionnaire distributed among all experts.

**Results:**

8 risk factors of gestational diabetes were significant in Iran. The analysis of experts' views showed that "History of GDM or disorder in glucose tolerance in pregnancy" is the most important risk factor for developing GDM (40.7%). The second and third most important risk factors were "History of macrosomia (infant birth weight > 4.1 Kg)" (20.2%) and" History of diabetes in first degree relatives" (10.7%).

**Conclusion:**

Suggesting screening based on the determined order of these risk factors can reduce the cost and stress in pregnant women. Also, it makes patient identifying faster. The healthcare sector can consider these priorities determined in experts' views to prevent gestational diabetes.

## Introduction

Gestational diabetes is developed in the pregnant women in whom pancreas function is not enough to overcome resistance to insulin (1). The outbreak of this complication has a significant increase during recent years (2). The prevalence of gestational diabetes has been estimated 3.4% in Iran (3). High level of mother’s blood sugar is followed by complications for mother and her baby (4). About 50% of the women suffered from gestational diabetes, will be suffered from type 2 diabetes during 5 years after pregnancy (5, 6). There is a relationship between increased blood sugar during pregnancy and children’s obesity at age of 5-7 years (7), infant macrosomia and mother's caesarean (4, 6).

Screening for treating gestational diabetes is an opportunity for preventing the complications of it (8). The main problem in public screening is cost effectiveness (9). This cost is an important issue for many Asian countries. According to a study conducted in 2005, the cost of a general screening in Iran has been calculated to be the US $2.50 for Glucose Challenge Test (GCT) and the US $7.50 for Glucose Tolerance Test (GTT) (10). The main issue in selective screening is the international disagreement on risk factors (11). Several international specialized groups have suggested using risk factors to identify the women at risk of gestational diabetes (12). The studies conducted in Iran have shown various factors as the most important risk factors for gestational diabetes (13-15).

One of the effective and appropriate methods for group decision-making to determine the most important factors and rank them is the Group Analytical Hierarchy Process (GAHP) (16). This method is one of the most famous techniques of Multi-Attribute Decision Making (MADM) presented by Saaty in 1970 for the first time. For decision-making, firstly, hierarchy structure of criteria and alternatives is created and then paired comparisons are performed among criteria. In calculating these comparisons, the weight of each criterion and their priorities are specified (17). 

In the last 10 years, using this method is clearly increasing in health care and medical decision-making. According to a systematic review conducted, this trend increased 20% from 2002 to 2016, which 12% of it is related to the last two years. This method is used in a wide range of medical and health care decision-making such as assessing and selecting care and treatment methods and assessing technology and policies of health care (18). Risk factors related to gestational diabetes are largely derived from studies on European populations(19), and a few studies have tested them in other populations (10). 

This research aims at deriving risk factors of gestational diabetes from literature and determining the importance of these risk factors in Iran according to the experts' views by GAHP.

## Materials and methods

**Questionnaire**

The questionnaire of the Analytical Hierarchy Process (AHP( was developed using a 9 points scale (Table I). In the AHP created on paired comparisons, for n criteria, the number of these comparisons is n(n-1)/2. Therefore according to the 8 criteria in this study, the total numbers of questions for paired comparisons among criteria were 28. 

**Procedure**

In this cross-sectional study, firstly, the papers related to the study of the prevalence and risk factors of gestational diabetes in Iran from 1992-2015 were reviewed. In this study, 27 papers related to the prevalence of gestational diabetes and its risk factors were extracted. By studying texts and Up to Date database, 10 risk factors for gestational diabetes were collected. Among these 10 items, the risk factors that have become significant based on studying literature in Iran were selected for performing the analysis process (1). 

This list includes 8 risk factors of gestational diabetes that have been presented in table II. The two risk factors of ethnicity and mother's weight at birth have not been examined in Iran and thus were omitted. In the process of AHP, to perform paired comparisons, the researches distributed questionnaire among all the population of research. The populations at this research were endocrinology experts of Mashhad, Iran. After following up in some phases, 11 experts completed the questionnaire. For each questionnaire, a paired comparisons matrix was formed and then the matrices were normalized using the following formula:

$\left(\left(\begin{array}{ccc} a_{11} & \cdots & a_{1n}\\ \vdots & \ddots & \vdots \\ \mathrm{n1} & \cdots & a_{\mathrm{nn}} \end{array}\right)\right)$

(1)

aij*=aij∑n1=1aij, j=1, 2,…, n

(2)

To calculate the consistency ratio of comparisons of each expert, consistency index (CI) of each matrix was calculated by the following question:

$\mathrm{CI}=\frac{\lambda_{\max}-n}{n-1}$

(3)

λ_max_ is the largest eigenvector of paired comparison matrix and n is the number of criteria. Then, Consistency Ratio (CR) was calculated using this formula: 

$\mathrm{CR}=\frac{\mathrm{CI}}{\mathrm{RI}}$

(4)

RI is consistency index obtained from the pared comparison matrix that has been generated randomly (Table III). In this study, the numbers of criteria are 8, so (*RI*) value is 1.41. Consistency Ratio means that there is an acceptable contradiction in response to the questions. According to the literature, If CR is <0.1 for any matrix, consistency of answers is acceptable. Two questionnaires have CR higher than 0.1 and were excluded from the rest of calculations. The 9 remained questionnaires having acceptable consistency were entered group calculation. In the next step, the weight of any risk factor was calculated according to each expert's views using the following equation:

wi=∑ni=1aijn

(5)

Then, the outlier data was identified based on Interquartile Range (IQR)= Q3-Q1; if a data was out of the range Q1-1.5 (IQR), Q3-1.5 (IQR), was identified as an outlier (20). Expert's views involving the outlier data were omitted. Then, to calculate the final weight of each risk factor, the remained views of experts were combined by Geometric Mean method. Prioritizing risk factors was performed according to the order of the calculated weights. 

**Ethical consideration**

The study received ethics approval from the Ethical Committee of Mashhad University of Medical Sciences, Mashhad, Iran, and all participants consented orally.

## Results

Based on studying the literature, the risk factors having a significant relationship with gestational diabetes in Iran following categorizing were: age above 30 years, number of previous pregnancies, obesity, family history of diabetes, history of previous unexplained perinatal loss or malformed infant birth, history of macrosomia infant birth, history of gestational diabetes or impaired glucose tolerance, glycosuria, history of infertility, hydraminus, history of caesarean in the previous and first childbirth. Among the 10 risk factors extracted from Up to Date, 8 risk factors were significant in Iran. For each of the 11 questionnaires returned by experts, paired comparisons matrix was created, normalized and its consistency ratio was calculated using formula 4 (Table IV). The responses related to DM4, DM5, greater than 0.1 and they were excluded from further calculations. For the remained 9 questionnaires, the weight of each index was calculated (Table V). 

Based on table V, the view of DM1 about index C3 (0.26%), and the view of DM3 about C7 (14%) were identified as an outlier. Because of this, the views related to DM1 and DM3 were omitted completely and the rest of calculations were performed according to the views of the 7 remained experts. Based on the data of table V, the risk factor of ''history of gestational diabetes or impaired glucose tolerance in previous pregnancies'' has the highest weight from the perspective of 7 experts. These risk factors have also the highest weight difference with other risk factors. The final matrix resulted from combining the paired comparisons matrix of any expert has been shown in table VI. The final weight of each index is derived from this matrix. Matrix data shows that based on the consensus of experts, the risk factor of ''history of gestational diabetes or impaired glucose tolerance in the previous pregnancies" have a 7.26 fold importance than the index of ''maternal age >25 years''. Also, this risk factor has a 7.23 fold importance than ''history of previous unexplained perinatal loss or birth of malformed infants''. 

The calculated weights and rank of each risk factors have been shown in table VII. The weight of each risk factor shows the rate of its effect on developing gestational diabetes. The risk factor of ''history of previous unexplained perinatal loss or birth of a malformed infant'', ''glycosuria at the first prenatal visit'', and ''maternal age >25 year'', by a trivial difference, have a least weight than other risk factors.

**Table I.** The 9-points scale used for the questionnaire of Analytical Hierarchy Process (17)

**9-points scale**	**State of comparing two risk factors**	**Explanation**
1	Equal importance	Criteria or alternative i is as important as j and or they have not priority than each other
3	Rather more important	Criteria or alternative i is a little more important than j
5	More important	Criteria or alternative i is more important than j
7	Much more important	Criteria or alternative i is much more important than j
9	Absolutely important	Criteria or alternative i is absolutely more important than j and is not comparable with j
2, 4, 6, 8		Show the intermediate values among the preferred values, for example, 8 expresses a higher importance than 7 and lower than 9 for i

**Table II:** The derived risk factors for analysis

**No**	**Risk factors of gestational diabetes**
1	History of gestational diabetes or impaired glucose tolerance in previous pregnancies
2	History of diabetes in family, especially in first-degree relatives
3	BMI*.=30 kg/m^2 ^before pregnancy or high weight gaining during pregnancy
4	Maternal age > 25 years
5	History of macrosomia infant birth (Weight >4.1 kg)
6	History of unexplained perinatal loss or a malformed infant birth
7	Glycosuria at the first prenatal visit
8	Medical conditions/setting related to the development of diabetes.

* Body Mass Index

**Table III:** Random Index for various values of n (matrix dimension

n	1	2	3	4	5	6	7	8	9	10
RI*	**0**	**0**	**0.58**	**0.9**	**1.12**	**1.24**	**1.32**	**1.41**	**1.45**	**1.49**

* Random Index (RI) is constant value for each n

**Table IV:** Consistency Ratio of responses of each expert

	**DM**_1_	**DM**_2_	**DM**_3_	**DM**_4_	**DM**_5_	**DM**_6_	**DM**_7_	**DM**_8_	**DM**_9_	**DM**_10_	**DM**_11_
**CR**	0.08	0.1	0.05	0.56	0.46	0.1	0.07	0.08	0.09	0.1	0.05

DM: Decision Maker

CR: Consistency Ratio

**Table V:** Weight of each risk factor in each expert's view

**index**	**W**_DM1_	**W**_DM2_	**W**_DM3_	**W**_DM6_	**W**_DM7_	**W**_DM8_	**W**_DM9_	**W**_DM10_	**W**_DM11_
History of gestational diabetes or impaired glucose tolerance in previous pregnancies (C1)	11%	42%	27%	41%	31%	41%	44%	35%	39%
History of diabetes in first degree relatives (C2)	5%	11%	3%	9%	10%	12%	7%	11%	17%
BMI*.=30 kg/m^2 ^before pregnancy or high weight gaining during pregnancy (C3)	26%	10%	12%	15%	11%	9%	9%	6%	7%
Maternal age > 25 years (C4)	6%	2%	2%	5%	3%	2%	2%	2%	3%
History of macrosomia infant birth (Weight *>4.1 kg) (C5)	14%	22%	20%	16%	34%	23%	14%	23%	13%
History of unexplained perinatal loss or a malformed infant birth (C6)	2%	4%	3%	4%	3%	4%	5%	3%	4%
Glycosuria at the first prenatal visit (C7)	2%	2%	14%	4%	4%	2%	4%	3%	3%
Medical condition/setting associated with development of diabetes (C8)	32%	7%	18%	6%	5%	7%	16%	18%	14%

* Body mass index

**Table VI:** The combined matrix of paired comparisons matrix of each expert

	**C**_1_	**C**_2_	**C**_3_	**C**_4_	**C**_5_	**C**_6_	**C**_7_	**C**_8_
C_1_	-	5.81	5.56	7.98	3.01	8.24	8.24	4.88
C_2_	0.17	-	1.35	4.55	0.5	4.13	3.51	1
C_3_	0.18	0.74	-	5.27	0.41	3.36	3.62	0.74
C_4_	0.13	0.22	0.19	-	0.2	0.39	0.62	0.27
C_5_	0.33	1.98	2.46	5.03	-	6.66	6.84	3.24
C_6_	0.12	0.24	0.3	2.56	0.15	-	2.1	0.33
C_7_	0.12	0.28	0.28	1.6	0.15	0.48	-	0.36
C_8_	0.2	1	1.35	3.65	0.31	3.06	2.77	-

**Table VII:** Calculated weights and ranking of risk factors for gestational diabetes

**Rank**	**Weight**	**Criteria**
1	40.7%	History of gestational diabetes or impaired glucose tolerance in previous pregnancies (C1)
2	20.2%	History of macrosomia infant birth (Weight >4.1 kg) (C5)
3	10.7%	History of diabetes in first-degree relatives (C2)
4	9.4%	BMI*=30 kg/m^2 ^before pregnancy or high weight gaining during pregnancy (C3)
5	9.3%	Medical condition/setting associated with development of diabetes (C8)
6	4%	History of unexplained perinatal loss or a malformed infant birth (C6)
7	3.1%	Glycosuria at the first prenatal visit (C7)
8	2.6%	Maternal age >25 years (C4)

* Body Mass Index

## Discussion

This research is conducted in order to determine the importance of risk factors for gestational diabetes in Iran by the Group AHP method. Among the risk factors derived based on studying the literature, the risk factor of ''history of gestational diabetes or impaired glucose tolerance in the previous pregnancies'' has the most importance in developing gestational diabetes than other risk factors. As the study by Huvinena showed, in spite of the healthier metabolic condition at first, non-obese women with the history of gestational diabetes had a higher rate of gestational diabetes outbreak (21). The healthcare providers should have a high sensitivity towards identifying the risk factors of gestational diabetes, especially the risk factor of the previous gestational diabetes (22). The experts' views in this study showed that glycosuria has a low importance in diagnosing gestational diabetes and the seventh rank among the eight risk factors. In the past, the test of glycosuria has had a weak sensitivity and characteristic. Recently, the guides of U.K. National Institute of Clinical Excellent have not recommended screening by this test (23).

In spite of many conducted studies (24-26), age was selected as a least important risk factor. In a systematic review and meta-analysis study by Jafari Shobeiri in 2015, the most important risk factors for gestational diabetes in Iran includes the history of gestational diabetes, history of family diabetes, BMI, the number of previous unexplained perinatal loss and number of the previous childbirth and history of having a macrosomic infant (3). Probably, changing lifestyle can be considered related to this result. In contrast, the study by Teh and coworkers by the method of logistic regression showed mother's age as the most important risk factor for gestational diabetes (27). 

The study on 924 pregnant women that was conducted by Shirazian and coworkers in 2009 showed that the risk of complications of gestational diabetes rises with increase of age>30, BMI>30, and history of family diabetes (28). In the most of the studies conducted in Iran, the same screening method and diagnostic criterion were not used in all studies (25, 29-31), or the sample size has been low in some studies. In 17 provinces of Iran, a study of prevalence and identifying risk factors for gestational diabetes has not been performed; As a result, a precise statistics of prevalence and its risk factors in the whole country is not available (32). In this study, the risk factors derived from the literature were compared with the risk factors of Up To Date. The other risk factors were omitted because of the mentioned reasons.

The results of this study were obtained based on experts' views and AHP, and according to researchers' studies, it seems that it is the first study on this topic. In most studies, the importance of risk factors has been determined by regression. AHP has been widely used in the healthcare sector (18). For example, prioritizing risk factors of obesity (33) and risk factors of Obstructive Sleep Apnea (34), the study by Pecchia and coworkers to derive users' s demands in CT (Computed Tomography) using the views of 5 experts (35), the study by Danner and coworkers based on the views of 7 experts and 12 patients aiming at eliciting patients' preferences for assessing health technologies (36), the study by Hilgerink using the views of 7 experts to assess the added value of the Twente Photo acoustic Mammoscope in breast cancer diagnosis (37), and the study by Suner for decision support in rectal cancer using the views of 5 experts can be referred to (38).

The required sample size is one of the discussions related to the AHP method. There is no subtle rule about calculation the sample size, but consensus and common agreement is that it does not need to large sample size (39). In this study, the questionnaires were distributed among all the research population and finally, 11 people responded. In AHP method, properly selecting the experts in the field of research is much more important than the number of them (39). The participants in this research were all Endocrinology Experts with a mean of 8.8 years of work experience, were in a close contact with the mothers suffered from gestational diabetes, and this causes more recognition of risk factors related to this disease and enhancement of results. 

The available limitation in conducting research is that only the views of Endocrinology Experts of Mashhad province are considered. Race and ethnicity as a risk factor have been stated in the valid scientific references, but so far no study has addressed the effect of this risk factor in gestational diabetes in Iran. However, because Mashhad is one of the metropolises of Iran in which peoples of other provinces live, this limitation cannot influence on the generalizability of the research results definitely. 

## Conclusion

Conducting a comprehensive study for examining the prevalence and identifying risk factors for gestational diabetes in Iran seems necessary. According to this fact that various ethnicities live in Iran, studying the difference of prevalence and risk factors among them is interesting. Given the board applications that the AHP has in the healthcare sector, using the results of this study by the physicians as criteria for identifying the pregnant women at risk and applying diagnostic methods is recommended. Also, to prevent gestational diabetes, the healthcare sector can consider these priorities determined in experts' views.
